# Adaptation and Validation of the Socio-Educational and Cultural Ambivalence Scale in the Mapuche School Context

**DOI:** 10.3390/bs16020272

**Published:** 2026-02-13

**Authors:** Enrique Riquelme Mella, Flavio Muñoz-Troncoso, Héctor Torres, Gloria Mora-Guerrero, Daniel Quilaqueo

**Affiliations:** 1 Departamento de Diversidad y Educación Intercultural, Education Faculty, Universidad Católica de Temuco, Temuco 4810296, Chile; eriquelme@uct.cl (E.R.M.); dquilaq@uct.cl (D.Q.); 2 Departamento de Educación e Innovación, Faculty of Education, Universidad Católica de Temuco, Temuco 4810296, Chile; 3 Faculty of Social Sciences and Arts, Universidad Mayor, Temuco 4801043, Chile; 4 International Observatory on School Climate and Violence Prevention (IOSCVP), 41004 Sevilla, Spain; 5 Campus Fernando May, Department of Educational Sciences, Faculty of Education and Humanities, Universidad del Bio-Bio, Chillán 3800708, Chile; htorres@ubiobio.cl; 6 Escuela de Psicología de la Facultad de Humanidades, Universidad de Santiago de Chile, Santiago 9170022, Chile; gloria.mora@usach.cl

**Keywords:** socio-educational ambivalence, psychometric validation, interculturality, mapuche education, epistemic pluralism

## Abstract

This study aimed to adapt and psychometrically validate the Socio-Educational and Cultural Ambivalence Scale (EASC) in the context of Chilean intercultural education, considering teachers, students, and parents/caregivers. Socio-educational ambivalence is defined as the coexistence of contradictory beliefs, emotions, and practices in the relationship between dominant school knowledge and Mapuche educational knowledge. Using a sequential mixed qualitative–quantitative design, we conceptually reviewed the original instrument and administered the adapted version to a sample of 739 participants (266 teachers, 286 students, and 183 parents/caregivers) from the regions of Biobío, La Araucanía, and Los Lagos. We proposed two six-factor scales: one shared by adults (teachers and parents/caregivers) and another with the same structure but fewer indicators for students. Confirmatory factor analysis (CFA) showed good model fit for both teachers and parents/caregivers (χ^2^ = 1100.85, df = 311, *p* < 0.001; RMSEA = 0.075; SRMR = 0.058; CFI = 0.934; TLI = 0.926) and students (χ^2^ = 378.546, df = 146, *p* < 0.001; RMSEA = 0.074; SRMR = 0.033; CFI = 0.978; TLI = 0.974). Composite reliability coefficients were ω = 0.702–0.974 for adults and ω = 0.749–0.948 for students. The results support factorial validity, internal consistency, and scalar invariance for the adult category of the instrument (teachers and parents/caregivers), confirming its usefulness for assessing epistemic and cultural tensions in intercultural educational contexts. The EASC contributes to the development of tools that foster a more plural, reflective, and context-sensitive understanding of education in Indigenous territories.

## 1. Introduction

Intercultural education in Chile has long faced the persistent challenge of articulating two epistemic universes: the dominant school knowledge of Western origin and the educational knowledge of Indigenous peoples, particularly that of the Mapuche (Quilaqueo et al., 2017; Walsh, 2010). In this context, the phenomenon of socio-educational and cultural ambivalence emerges as a key category for understanding the cognitive, emotional, and ethical tensions experienced by teachers, students, and families as they interact within the school setting. This ambivalence reflects the coexistence of contradictory cultural orientations: on one hand, adherence to the values of modern schooling, and on the other, the persistence of practices, beliefs, and rationalities rooted in Mapuche knowledge systems (Gasché, 2013; Tabboni, 2007; Quilaqueo & Torres, 2022).

From a theoretical standpoint, ambivalence is understood as a structural disposition of social actors who oscillate between opposing norms or values (Simmel, 1971; Merton, 1968; Elias, 1994). In the educational field, this category becomes particularly relevant in culturally diverse settings, where pedagogical relations are shaped by colonial hierarchies, symbolic inequalities, and identity-based tensions that strain the dialogue between knowledge systems (Bertely & Gasché, 2011; Tubino, 2007). In Chile, the schooling of the Mapuche people reproduces mechanisms of sociocultural domination rooted in the monocultural epistemology of modern scientific rationality (Santos, 2009; Quilaqueo et al., 2020). This rationality, according to Olivé (2009), has established an epistemological monism that excludes or subordinates Indigenous forms of knowledge, reinforced by the content of the national curriculum and teacher education programs.

The concept of educational ambivalence refers to the coexistence of opposing principles, beliefs, and emotions within individuals and institutions involved in teaching and learning processes. Building on Simmel (1971), Merton (1968), and Elias (1994), ambivalence represents the dual orientation of human behavior between defending individual life and participating in collective life. In the school environment, this ambivalence is expressed in the internal contradictions experienced by teachers and students regarding the values, symbols, and expectations that coexist in culturally diverse contexts. It is not merely indecision or cognitive conflict but a structural tension between epistemic rationalities—one dominant, the other subordinated—that operate simultaneously within the educational field (Gasché, 2013; Tubino, 2007). In intercultural contexts, this ambivalence takes two complementary forms: socio-educational ambivalence and cultural ambivalence.

Socio-educational ambivalence refers to the coexistence of frameworks for valuing knowledge—school-based and Indigenous—that shape the ways in which educational content is taught, learned, and evaluated. It should not be understood as a temporary contradiction but rather as an internal structure of pedagogical thought and action in contexts of sociocultural domination that hinder intercultural engagement. Following Simmel’s (1971) classical reading, ambivalence is an inherent feature of social life: individuals act under opposing impulses—individualism and collectivism, permanence and change, authority and autonomy—which are simultaneously expressed in daily experience. Merton (1968) expanded this notion by arguing that social roles generate incompatible demands that individuals must balance through adaptive strategies.

From this perspective, educational ambivalence produces the cognitive, affective, and value-based tensions that permeate teaching and learning processes when different knowledge systems coexist. It represents a form of structural dissonance that emerges both from inter-societal conflict and from the simultaneous internalization of opposing cultural rationalities: an instrumental and universalist rationality associated with modern school knowledge, and a community-based, situated rationality linked to Indigenous and family knowledge systems (Gasché, 2013).

Among teachers working in schools with Mapuche student populations, this ambivalence may manifest as difficulty in recognizing or incorporating educational content—such as the Mapuzugun language—into their pedagogical practice. For students, ambivalence often emerges as a fragmented identity experience: While they learn to navigate the expectations of the school system, they must also resist or reinterpret narratives that render invisible their roots and family knowledge. This dynamic creates a constant field of tension between who they are expected to be and who they are within their cultural experience (Bertely & Gasché, 2011).

Cultural ambivalence, more affective and symbolic in nature, describes the tension experienced by individuals who participate in different cultural systems and must reconcile diverse identity, spiritual, and community-based loyalties (Tabboni, 2007; Schütz, 1976). It is conceptualized as an internal state of tension between incompatible value systems and expectations, where individuals experience simultaneous acceptance and resistance (Tabboni, 2007; Gasché, 2013). In the Mapuche context, this ambivalence manifests as an oscillation between loyalty to family knowledge and the need to adhere to models of “Western success.” Thus, a permanent space of identity negotiation emerges (Riquelme et al., 2024), marked by the coexistence of opposing normative ideals, such as the promotion of cultural diversity and adherence to universal standards of knowledge (Elias, 1994; Tabboni, 2007).

### The Need for an Instrument to Measure Socio-Educational Ambivalence

From a psychological perspective, this condition expresses both conflict and adaptive potential, understood as the capacity of individuals to articulate divergent cultural meanings and generate creative syntheses. For this reason, instruments designed to assess ambivalence do not aim to pathologize it, but rather to measure its structure and directionality.

In epistemic terms, ambivalence does not imply inconsistency; instead, it reflects the possibility of recognizing the coexistence of multiple rationalities within educational processes (Fornet-Betancourt, 2018; Walsh, 2010). From this viewpoint, ambivalence is also a sign of cultural resistance, as it expresses the effort of individuals to maintain their own frameworks of reference amid the homogenizing pressures of schooling. Understanding it, therefore, requires an approach that integrates the psychological, sociocultural, and political levels, articulating the affective dimensions of conflict with the institutional structures that sustain it (Quilaqueo & Torres, 2022).

We thus consider ambivalence as lying at the core of the epistemic tension between dominant school knowledge and subordinated Mapuche educational knowledge, identifying within it a key to understanding the obstacles faced by education in culturally diverse contexts.

Despite its theoretical relevance, socio-educational ambivalence has been scarcely operationalized in educational research. Most studies on intercultural education have centered on qualitative descriptions of teacher discourse or practice, without developing instruments capable of empirically assessing epistemic and cultural tensions in the classroom. This gap limits cross-context comparisons and constrains the development of evidence-based public policies.

The development of the Socio-Educational and Cultural Ambivalence Scale (EASC) responds to a twofold need. First, it seeks to create a psychometric instrument that translates the conceptual categories of epistemological pluralism into observable indicators while maintaining theoretical fidelity to the model of double educational rationality. Second, it aims to provide a tool capable of assessing the presence and levels of ambivalence among both teachers and students, enabling comparative analyses across regions, educational levels, and cultural groups.

The design of the scales draws upon this theoretical matrix to measure cultural orientations toward the school system, the valuation of Mapuche knowledge, and perceptions of the educational system as a factor of assimilation or integration, thereby allowing the construction of educational ambivalence profiles associated with acculturation strategies.

The scale was conceived to uncover the nature of socio-educational and cultural ambivalence that strains the dialogue between Mapuche educational knowledge and school-based knowledge. It therefore constitutes an essential methodological step toward consolidating an empirical field of critical intercultural education in Chile.

From an epistemological standpoint, having such a tool makes it possible to advance toward a sociology of educational ambivalences—a perspective that recognizes the plurality of meanings, resistances, and negotiations shaping pedagogical practice in culturally diverse territories. From an applied perspective, it enables the identification of ambivalence patterns that may inform teacher training processes, curricular redesign, and psycho-educational support for Indigenous students.

In summary, exploring socio-educational ambivalence through a valid and reliable instrument is both a scientific and an ethical endeavor. It entails not only measuring a psychological variable but also capturing a culturally situated relational dynamic that reveals the tensions—and potential forms of recognition—between the epistemes that coexist within Chilean schools.

## 2. Materials and Methods

### 2.1. Approach and Study Design

An instrumental design was developed to adapt and psychometrically validate the Socio-Educational and Cultural Ambivalence Scale (EASC) in its versions for students (EASC-E), teachers (EASC-D), and parents/caregivers (EASC-P). The scale was conceived to measure the coexistence of contradictory cultural orientations within intercultural educational processes.

### 2.2. Participants

A total of 739 individuals participated, ranging in age from 10 to 72 years (M = 30.48; SD = 15.6). Of the sample, 62.9% identified as women, 36.1% as men, and 0.9% did not report their gender. Regarding ethnic identification, 62.7% self-identified as Mapuche and 37.3% as non-Mapuche. Participants were distributed across three school roles: 36% teachers, 38.8% students, and 25.2% parents/caregivers from primary and secondary schools in the regions of Biobío (2.2%), La Araucanía (83.4%), and Los Lagos (14.5%).

Teacher ages ranged from 23 to 72 years (M = 41.83; SD = 10.39); parents/caregivers ranged from 19 to 60 years (M = 40.54; SD = 9.16); and students ranged from 10 to 18 years (M = 13.55; SD = 1.98). Gender and ethnic ancestry distributions for each group are presented in Table 1.

This sample size met recommended criteria for factor analyses, including at least five to ten cases per item (Nunnally & Bernstein, 1995; Lloret-Segura et al., 2014), ensuring parameter stability and reliability in model estimation. School selection was based on intercultural relevance, requiring that at least 10% of enrolled students self-identify as Mapuche, according to records from the Ministry of Education (Mineduc) and the 2017 National Census (INE, 2018). For the teacher group, inclusion criteria required being employed in schools with a significant enrollment of Mapuche students. For the student group, inclusion criteria were (a) self-identification as Mapuche and (b) being enrolled between 8th grade and 12th grade.

### 2.3. Instruments

The Socio-Educational and Cultural Ambivalence Scale (EASC) is grounded in a theoretical framework that integrates two conceptual traditions: (a) the model of double educational rationality (Quilaqueo et al., 2016; Quilaqueo et al., 2022), which conceives the coexistence of two epistemic systems in tension—the modern school rationality and the Mapuche community rationality—and (b) the theoretical approach to educational ambivalence as cognitive and affective tension in response to conflicting values and knowledge systems (Gasché, 2013; Tabboni, 2007).

Socio-educational and cultural ambivalence is expected to manifest differently depending on the actor’s role within the school institution. While Mapuche students experience this ambivalence as an identity- and emotion-based tension in relation to school, parents/caregivers encounter it as a dissonance between family educational values and the norms of the formal school system. Consequently, two complementary and conceptually equivalent instruments were developed, designed to capture the perceptions and experiences of both groups from their respective positions within the educational community.

Thus, the design of the scales followed a parallel logic: maintaining conceptual equivalence across instruments while adapting language, level of abstraction, and item complexity to the characteristics of each target group. Both questionnaires were originally developed in Spanish and underwent linguistic review in Mapuzugun to ensure cultural and semantic adequacy. Given the importance of elderly relatives and extended family (e.g., grandparents) in Mapuche culture, we use parents/caregivers interchangeably to reflect diverse family configurations. The same scale was administered to all parent/caregiver respondents. Parent/caregiver and teacher scales are identical in structure and items, with minimal wording adjustments only where role-specific language was necessary.

*a.*  *Conceptual Exploration and Item Construction*

Instrument development was grounded in the framework of double educational rationality and was additionally informed by prior qualitative evidence developed by the authors. Specifically, an independent qualitative study (currently under review) explored ambivalence as expressed in both the family sphere and the educational/school sphere within intercultural schooling settings, using semi-structured interviews and participatory activities with educational actors and families. The categorical analysis conducted in that study identified recurring thematic clusters and semantic cores that conceptually align with the six dimensions proposed in the present manuscript and guided the initial conceptual specification of the model and item allocation. Importantly, this qualitative study did not constitute a qualitative phase of the current psychometric validation; rather, it represents a separate line of inquiry that provided contextual and semantic grounding for the a priori structure subsequently tested here. All three versions share the same factorial structure, although item wording was adapted to respondents’ roles and contexts (see Appendix A). The teacher (EASC-D) and parent/caregiver (EASC-P) versions each contain 36 items, while the student (EASC-E) version contains 25 items, rated on a five-point Likert scale (1 = strongly disagree to 5 = strongly agree).

The scale is organized into six dimensions that explore the relationship between educational practices, local knowledge, and cultural recognition.

*1. Integration of Contextual and Cultural Knowledge:* Evaluates the incorporation of Mapuche cultural and contextual knowledge into the school curriculum. High scores indicate meaningful integration between local and academic knowledge; low scores reflect the dominance of Western knowledge.

Examples:

EASC-E: “E_10 I like when we use examples from the Mapuche community to understand school subjects.”

EASC-P: “P_10 We consider it important for the school to incorporate knowledge from the territory and Mapuche culture.”

EASC-D: “D_10 I strive to include local Mapuche knowledge when teaching school content.”

*2. School–Community Linkages and Knowledge Mobilization:* Assesses collaboration between school, family, and community in transmitting traditional knowledge.

High scores indicate active, reciprocal links; low scores suggest disconnection or symbolic participation.

Examples:

EASC-E: “E_13 I enjoy when school activities involve the Mapuche community.”

EASC-P: “P_13 We gladly participate when the school organizes activities with the community.”

EASC-D: “D_13 I value collaboration with Mapuche families in educational and cultural projects.”

*3. Equal Treatment and Recognition of Cultural Diversity:* Examines equitable treatment and recognition of cultural diversity within the school. High scores indicate inclusion and respect; low scores reflect invisibilization or disregard of differences.

Examples:

EASC-E: “E_15 Teachers value the different cultures of students.”

EASC-P: “P_15 The school recognizes and respects the culture of every family.”

EASC-D: “D_15 I strive for all my students’ cultures to be equally valued in the classroom.”

*4. Cultural Identity and Resistance to Homogenization:* Assesses the preservation of Mapuche identity in the face of homogenizing pressures within the school system. High scores indicate pride and continuity; low scores reflect cultural loss or displacement.

Examples:

EASC-E: “E_17 I am proud of my culture and I enjoy learning about it.”

EASC-P: “P_17 We are concerned that the school may weaken our children’s cultural identity.”

EASC-D: “D_17 Teaching should strengthen Mapuche students’ cultural identity.”

*5. Intercultural Education and Deepening of Traditional Knowledge:* Evaluates the depth with which Mapuche knowledge is taught, avoiding folklorization. High scores indicate substantive teaching; low scores reflect superficial or symbolic practices.

Examples:

EASC-E: “E_20 It is important to learn what Mapuche people know about plants and animals.”

EASC-P: “P_20 We value when the school teaches traditional knowledge beyond celebrations.”

EASC-D: “D_20 I seek to meaningfully incorporate traditional Mapuche knowledge into the curriculum.”

*6. Participation in Cultural Ceremonies and Hybrid Spaces:* Assesses participation in Mapuche ceremonies as spaces for cultural encounter and hybridization. High scores indicate meaningful and respectful participation; low scores reflect absence or trivialization.

Examples:

EASC-E: “E_23 I enjoy participating in cultural ceremonies at school.”

EASC-P: “P_23 We consider it important for schools to include Mapuche ceremonies as part of learning.”

EASC-D: “D_23 Participation in cultural ceremonies strengthens intercultural respect among students.”

#### Validation

The adaptation process followed international guidelines for test construction and validation in multicultural contexts (Muñiz et al., 2013), encompassing the following stages: (a) Conceptual review: The research team examined theoretical and empirical literature on ambivalence, interculturality, and educational rationalities to refine the scale dimensions. (b) Linguistic–cultural review: Semantic appropriateness of items for teacher and student populations was assessed, adapting expressions to the cultural and linguistic register of the Mapuche context. (c) Expert judgment: Experts in intercultural education, psychometrics, and sociology of education evaluated item clarity, relevance, and representativeness.

### 2.4. Procedure

The study was carried out in coordination with the participating schools. The scale was administered in person during school hours with prior authorization from the school principals. Each participant received written information about the study’s objectives and procedures, signing informed consent or assent as appropriate, in accordance with ethical guidelines for educational research (Sánchez, 1992; Aréchiga, 2004). Administration was conducted collectively, in groups of 15 to 30 participants, under the supervision of a trained researcher and an assistant. The average duration was 25 min. Data were recorded anonymously and coded in a secure database, ensuring confidentiality and participant integrity. The process also included the return of partial results to participating school communities, as part of the ethical and reciprocity commitments of the Fondecyt project.

### 2.5. Data Analysis

Although exploratory factor analysis (EFA) is commonly used in the early phases of scale development, we did not conduct a traditional EFA in the present study because the EASC was not designed to derive its dimensionality from a purely inductive statistical procedure. Instead, the scale was conceived as a theoretically grounded instrument with an a priori six-factor structure, consistent with the framework of double educational rationality and intercultural schooling. Moreover, this a priori specification was strengthened by prior qualitative categorical evidence produced by the authors in an independent study currently under review, which explored ambivalence in both family and educational spheres and identified thematic clusters that conceptually align with the six dimensions tested here. Importantly, this qualitative work should not be interpreted as a preliminary qualitative phase of the current psychometric study; rather, it represents a separate line of inquiry that contributed additional contextual and semantic grounding for the proposed structure.

Accordingly, we adopted confirmatory factor analysis (CFA) as the primary approach to evaluate the hypothesized factorial structure, test dimensionality, and examine item allocation across the six factors. In terms of item allocation, the adult versions retained 36 items distributed across the six dimensions, whereas the student version retained 25 items across the same dimensions but with fewer indicators. Given these differences in the number of indicators and the linguistic adaptation required for younger participants, analyses were conducted separately for (1) students and (2) adults. Additionally, measurement invariance analyses were used to examine whether the adult model functioned equivalently across teacher and parent/caregiver groups.

Because the student scale does not contain the same number of items as the teacher and parent/caregiver versions, the analyses were conducted independently for (1) students and (2) teachers and parents/caregivers.

Normality was examined for all items to select the appropriate estimators for subsequent analyses. Confirmatory factor analysis (CFA) was conducted using the robust weighted least squares estimator (ULSMV) (Brown, 2015; Muthén & Muthén, 2017). To evaluate model fit, the following indices were examined: chi-square and chi-square/df ratio; root mean square error of approximation (RMSEA); standardized root mean square residual (SRMR); Comparative Fit Index (CFI); Tucker–Lewis Index (TLI). In general, a model is considered to have adequate fit when χ^2^/df ≤ 3, RMSEA < 0.08 (ideally < 0.05), SRMR < 0.08, and CFI/TLI ≥ 0.90 (preferably ≥ 0.95) (Hu & Bentler, 1999; Kline, 2016; Byrne, 2016).

Regarding internal parameters, standardized factor loadings (λ) ≥ 0.40 were considered acceptable, although loadings ≥ 0.60 are desirable as they indicate an adequate representation of the item within its factor (Hair et al., 2019). Composite reliability (CR) should exceed 0.70, and average variance extracted (AVE) should reach at least 0.50, indicating convergent validity and internal consistency (Fornell & Larcker, 1981).

Discriminant validity was examined using the Fornell–Larcker criterion, comparing shared variance across constructs with variance extracted. Since discriminant validity was not achieved, a second-order factor model was tested, provided it met theoretical and statistical criteria (Varela et al., 2006). High standardized loadings (>0.90) between first- and second-order factors are not considered problematic when the model is well-identified and theoretically coherent (Hair et al., 2019; Kline, 2016).

A second phase examined measurement invariance between teachers and parents/caregivers. Invariance analysis assesses whether the factorial structure is equivalent across groups. Three hierarchical levels were tested: (a) configural invariance, verifying the same factor structure; (b) metric invariance, testing equality of factor loadings; and (c) scalar invariance, testing equality of intercepts or thresholds.

Invariance is supported when the decrease in CFI is ≤0.01 and the increase in RMSEA is ≤0.015 (Cheung & Rensvold, 2002; Chen, 2007). Normality analyses were conducted using SPSS v.26 (IBM Corp, 2020). CFA and invariance analyses were performed with Mplus v.8.8 (Muthén & Muthén, 2024). AVE, reliability, and convergent and discriminant validity were calculated using Microsoft Excel v.16 (Microsoft, 2023).

### 2.6. Ethical Considerations

The research was approved by the Ethics Committee of the Catholic University of Temuco (Ethical Res. No. 2023-047). The principles of voluntariness, confidentiality, anonymity, and community return of results were ensured, in accordance with the guidelines of intercultural ethics and respect for Mapuche knowledge (Tubino, 2007; Fornet-Betancourt, 2018)

## 3. Results

The Kolmogorov–Smirnov test indicated that the distribution of the data does not conform to normal (*p* < 0.05). Excellent goodness-of-fit indices were found for both the teacher and parent scale (χ^2^ = 957.33, df = 302, *p* < 0.001; RMSEA = 0.069; SRMR = 0.052; CFI = 0.946; TLI = 0.937) and the student scale (χ^2^ = 358.63, df = 137, *p* < 0.001; RMSEA = 0.075; SRMR = 0.031; CFI = 0.979; TLI = 0.974). The standardized loads of all items are greater than 0.4, which can be seen in the structure of the scales in Table 2 and Table 3.

In Table 4 and Table 5, it can be seen that the scales show good reliability indices and convergent validity. Likewise, it is appreciated that there is insufficient evidence in favor of discriminant validity between the latent variables.

**Figure 1:**
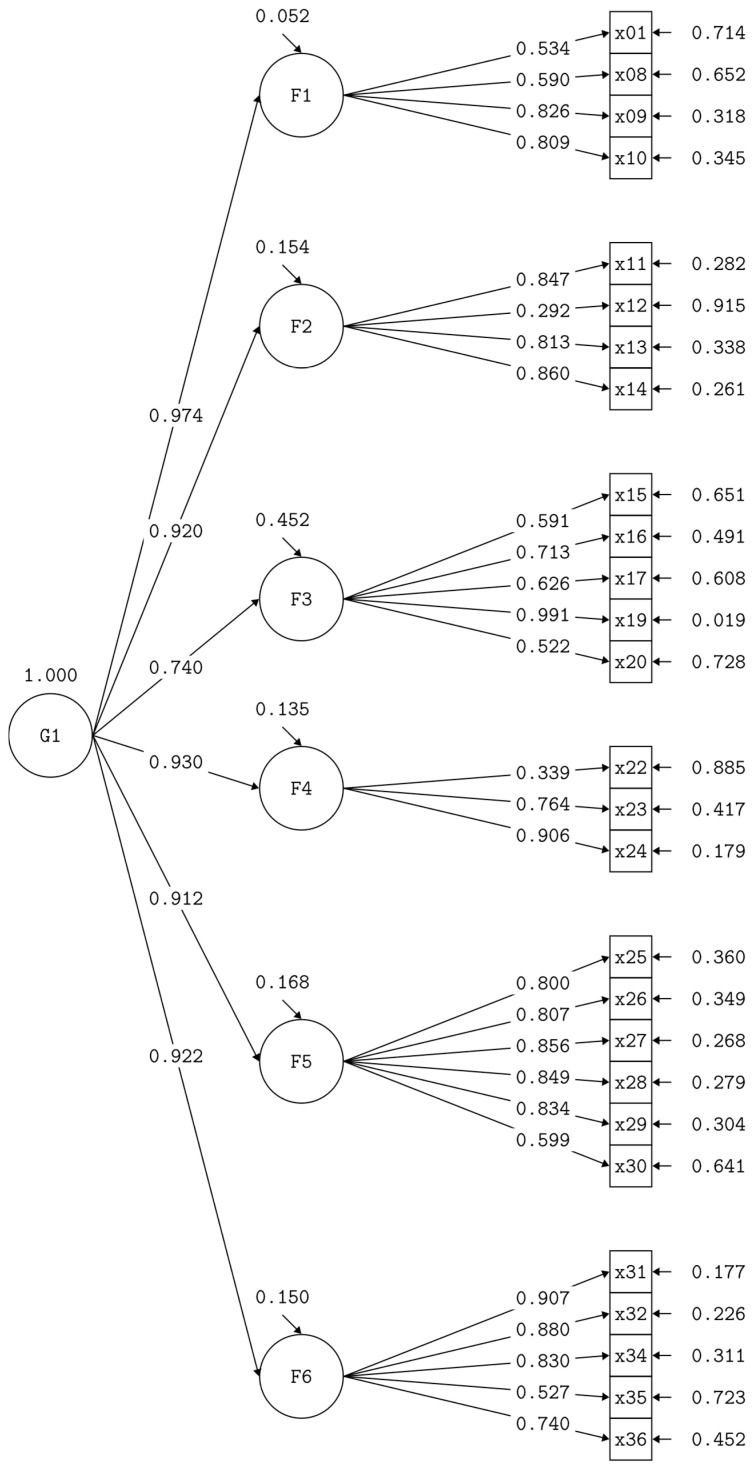
Second-order factor model, teacher and parent scale. Source: authors’ elaboration.

**Figure 2:**
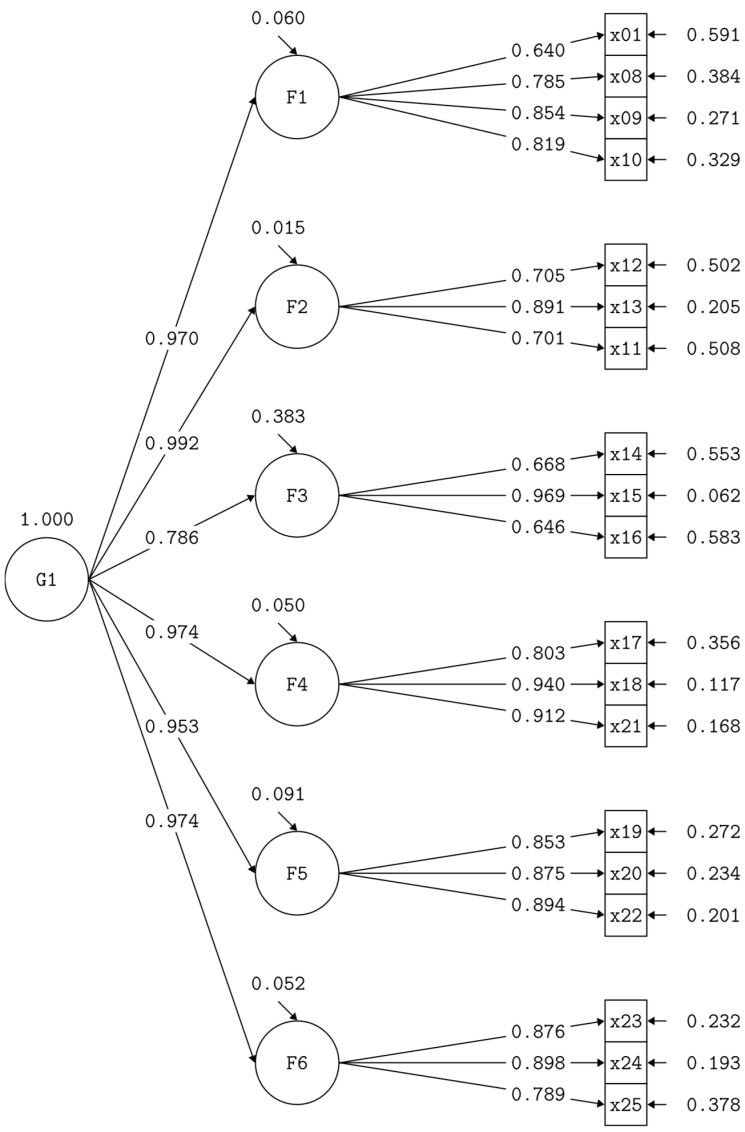
Second-order factor model, student scale. Source: authors’ elaboration.

Considering that there was no evidence of discriminant validity, a model with a second-order factor was proposed for each scale (Figure 1 and Figure 2). The model presented a good fit to the data for both the teacher and parent scale (χ^2^ = 1100.85, df = 311, *p* < 0.001; RMSEA = 0.075; SRMR = 0.058; CFI = 0.934; TLI = 0.926) and the student scale (χ^2^ = 378.546, df = 146, *p* < 0.001; RMSEA = 0.074; SRMR = 0.033; CFI = 0.978; TLI = 0.974).

### Invariance of Measurement

The proposed model showed a good fit to the data for both the group of teachers (χ^2^ = 606.53, df = 302, *p* < 0.001; RMSEA = 0.062; SRMR = 0.054; CFI = 0.957; TLI = 0.950) and the group of parents (χ^2^ = 617.87, df = 302, *p* < 0.001; RMSEA = 0.075; SRMR = 0.062; CFI = 0.946; TLI = 0.937). Scalar invariance was achieved for the role category (teachers and parents), which is evidenced in Table 6.

## 4. Discussion

The results obtained in the validation of the Socio-Educational and Cultural Ambivalence Scale (EASC) confirm both the theoretical relevance and the empirical robustness of the construct within the context of intercultural education. The six-factor structure showed satisfactory fit indices across the three versions of the scale (teachers, parents/caregivers, and students), indicating the stability of the double educational rationality model (Quilaqueo et al., 2016) as an explanatory framework for the cognitive, affective, and value-based tensions that shape intercultural school experiences.

First, the internal consistency of the scales and the high ordinal reliability (ω ranging from 0.73 to 0.91) demonstrate that socio-educational ambivalence can be measured with precision and compared across different groups within the school community. These findings provide empirical support for the hypothesis that ambivalence constitutes a structural disposition rather than an accidental or merely contextual phenomenon (Simmel, 1971; Tabboni, 2007). The confirmation of a second-order factor additionally suggests the presence of a latent overarching dimension—epistemic ambivalence—that encompasses the various manifestations of cultural tension between school knowledge and Mapuche knowledge.

Second, the absence of discriminant validity among the dimensions indicates a significant interdependence between the cognitive and affective components of the phenomenon. Theoretically, this reinforces the idea that practices of knowledge integration, recognition of cultural diversity, and participation in ceremonial spaces do not operate in isolation but rather as complementary expressions of a shared intercultural meaning structure (Gasché, 2013; Fornet-Betancourt, 2018). Consequently, the EASC not only measures attitudes but also allows for inferences about cultural relational modes and identity orientations in the context of schooling.

The evidence of scalar invariance between teachers and parents/caregivers is particularly relevant. This finding confirms that both groups interpret the evaluated constructs equivalently, enabling legitimate comparisons between school actors. The alignment in the factorial structure suggests that ambivalence is not an individual trait but a relational phenomenon emerging from the interaction of educational rationalities in tension (Walsh, 2010; Quilaqueo & Torres, 2022). From an intercultural perspective, this convergence underscores the need to understand ambivalence as a form of symbolic resistance and identity negotiation in response to the epistemological monism of the Chilean school system (Santos, 2009; Olivé, 2009).

Additionally, the high correlations between the dimensions related to the integration of contextual knowledge and deep intercultural education reflect a positive disposition toward knowledge dialogue, consistent with the integration strategy described by Berry (1997, 2017). However, the coexistence of items oriented toward assimilation or separation suggests that the school continues to be a space of unresolved cultural ambivalence: actors recognize the importance of Mapuche knowledge while simultaneously reproducing the legitimacy criteria of Western knowledge.

These findings allow ambivalence to be interpreted as an indicator of transition between educational models. Among teachers and parents/caregivers, it expresses the tension between institutional expectations of the national curriculum and community aspirations for cultural recognition; among students, it reflects the effort to articulate a bicultural identity within school structures that still privilege academic universalism. Thus, ambivalence becomes a marker of the epistemic inequalities that persist in Chilean education, as well as a potential site for pedagogical transformation.

Ultimately, this study confirms that socio-educational ambivalence should not be conceived as a deficit in cultural coherence but rather as an adaptive and critical response to educational contexts characterized by epistemic hegemony. In this sense, the EASC contributes significantly to the fields of intercultural psychology and the sociology of education by offering an empirical tool capable of translating into observable dimensions the symbolic and affective tensions that structure pedagogical experiences in Indigenous territories.

The study presents several limitations that must be acknowledged. First, although the sample was large and territorially diverse, it was concentrated in three regions of southern Chile, which limits the generalizability of the results to other Indigenous groups or urban areas with lower Mapuche representation. Second, the cross-sectional nature of the design prevents the assessment of the temporal stability of socio-educational ambivalence; thus, future studies should incorporate longitudinal measurements to observe how it evolves throughout the school trajectory.

Another limitation concerns the potential influence of social desirability bias, particularly among teachers, whose responses may have been influenced by institutional discourses on interculturality rather than by actual practices. Furthermore, the lack of discriminant validity among dimensions suggests the need to revise the wording of some items to increase empirical independence across factors and strengthen the structural validity of the model. Finally, the linguistic–cultural adaptation to Mapuzugun still requires a deeper semantic validation process to ensure full comprehension of the items in bilingual or community-based contexts.

It is recommended that the EASC be applied in other intercultural educational contexts in Latin America—such as Quechua, Aymara, or Guaraní communities—to explore the universality of the construct and compare forms of socio-educational ambivalence across different sociocultural configurations.

From a methodological standpoint, future research should develop longitudinal and multigroup invariance studies to identify trajectories of change in intercultural orientations among teachers and students, as well as the institutional conditions that foster or inhibit epistemic integration. In applied terms, the EASC can serve as a diagnostic tool in teacher training programs, supporting the identification of cultural tensions and guiding context-sensitive pedagogical support processes.

Finally, consolidating an empirical field around educational ambivalence opens an interdisciplinary research agenda that integrates psychometrics with the anthropology of education, discourse analysis, and public policy evaluation. The scale validated here represents a significant step toward constructing an epistemically plural education system in which cultural differences are not translated into hierarchies but rather into the foundations of a more just, reflective, and transformative dialogue of knowledge.

### Limitations

Several limitations of the present study should be acknowledged, as they delimit the interpretation of the results and open directions for continued research on socio-educational and cultural ambivalence in intercultural school contexts. First, the sample was regionally concentrated in a specific territory of southern Chile, characterized by particular configurations of Mapuche–non-Mapuche coexistence, schooling experiences, and sociohistorical dynamics. While this context is highly relevant for examining ambivalence as a situated socio-educational phenomenon, it may restrict the generalizability of the findings to other regions or intercultural settings with different demographic compositions, institutional conditions, and sociopolitical backgrounds.

Second, the study employed a cross-sectional design, which does not allow for the assessment of temporal stability. Although the CFA results support the factorial structure at a single time point, the absence of longitudinal evidence limits conclusions regarding the stability of the construct across school years and developmental stages. Establishing test–retest reliability and evaluating longitudinal invariance remain pending tasks.

Third, the instrument relies on self-report data, which may be vulnerable to social desirability bias, especially given the cultural sensitivity of the topics addressed (e.g., interethnic relations, recognition of cultural differences, and educational expectations). Participants may provide responses aligned with perceived normative positions or institutional expectations, potentially attenuating the variability of responses in specific dimensions.

Fourth, the CFA results indicated moderate-to-high intercorrelations among some factors, particularly in the adult model. Although conceptual proximity across dimensions is theoretically plausible in socio-educational constructs grounded in interrelated belief systems, this pattern may also reflect partial overlap and therefore calls for further examination of discriminant validity. Future evaluations could test alternative representations of the construct (e.g., higher-order or bifactor structures) and incorporate additional external criteria to clarify the distinctiveness and boundaries of each dimension.

Finally, while the scale was developed through procedures intended to ensure contextual adequacy, ambivalence is a construct with substantial linguistic and cultural complexity, and subtle differences in wording across respondent roles (teachers vs. parents/caregivers) and developmental levels (students) may introduce nuances in interpretation that are not fully captured by model fit indices alone. This underscores the need for deeper semantic and cultural validation, including qualitative approaches that explore how key terms are understood and negotiated across groups.

These limitations do not diminish the contribution of the present findings, but rather delineate a clear research agenda. Future studies are encouraged to extend validation efforts across territories and educational settings, incorporate longitudinal designs to assess stability and change, integrate strategies to mitigate social desirability, and further strengthen discriminant and semantic validity using complementary analytic models and culturally grounded qualitative procedures. Advancing along these lines will consolidate the EASC as a robust tool for understanding socio-educational and cultural ambivalence and for supporting evidence-informed intercultural educational practices.

## Figures and Tables

**Table 1:** Gender and ethnic ancestry distribution by role.

Role	Category	Group	Frequency	Percentage	Cumulative Percentage
Teacher	Gender	Men	75	28.2	28.2
		Women	187	70.3	98.5
		No response	4	1.5	100.0
		Total	266	100.0	
	Ancestry	Mapuche	79	29.7	29.7
		Non-Mapuche	187	70.3	100.0
		Total	266	100.0	
Student	Gender	Men	137	47.7	47.7
		Women	147	51.2	99.0
		No response	3	1.0	100.0
		Total	287	100.0	
	Ancestry	Mapuche	225	78.4	78.4
		Non-Mapuche	62	21.6	100.0
		Total	287	100.0	
Parents/Caregivers	Gender	Men	55	29.6	29.6
		Women	131	70.4	100.0
		No response	0	0.0	—
		Total	186	100.0	
	Ancestry	Mapuche	159	85.5	85.5
		Non-Mapuche	27	14.5	100.0
		Total	186	100.0	

Source: authors’ elaboration.

**Table 2:** Structure and factorial load scale—teachers and parents.

Factor	Number of Indicators	Min λ	Max λ
F1	4	0.534	0.823
F2	4	0.293	0.846
F3	5	0.520	0.983
F4	3	0.336	0.908
F5	6	0.595	0.856
F6	5	0.529	0.907

Source: authors’ elaboration.

**Table 3:** Structure and factor load scale—students.

Factor	Number of Indicators	Min λ	Max λ
F1	4	0.627	0.835
F2	3	0.690	0.870
F3	3	0.646	0.968
F4	3	0.803	0.940
F5	3	0.854	0.894
F6	3	0.789	0.898

Source: authors’ elaboration.

**Table 4:** Reliability and convergent validity of the teacher and parent scale.

	AVE	ω	F1	F2	F3	F4	F5	F6
F1	0.492	0.789	0.702					
F2	0.551	0.815	0.974	0.743				
F3	0.501	0.826	0.773	0.771	0.708			
F4	0.508	0.732	0.888	0.816	0.633	0.712		
F5	0.633	0.911	0.871	0.812	0.584	0.923	0.796	
F6	0.623	0.889	0.838	0.790	0.720	0.841	0.894	0.789

Source: authors’ elaboration. Note: The diagonal shows the square root of the AVE, and below the diagonal are the correlations between the factors.

**Table 5:** Reliability and convergent validity scale—students.

	AVE	ω	F1	F2	F3	F4	F5	F6
F1	0.561	0.864	0.749					
F2	0.617	0.760	0.899	0.785				
F3	0.600	0.813	0.805	0.765	0.775			
F4	0.787	0.917	0.947	0.950	0.765	0.887		
F5	0.765	0.907	0.936	0.930	0.753	0.949	0.875	
F6	0.732	0.891	0.948	0.899	0.747	0.943	0.922	0.856

Source: authors’ elaboration. Note: The diagonal shows the square root of the AVE, and below the diagonal are the correlations between the factors.

**Table 6:** Measurement Invariance Across Teacher and Parent Groups.

	χ^2^	Gl	*p*-Value	RMSEA	CFI	TLI	Δ CFI	Δ RMSEA
Configural	1120.465	604	<0.001	0.067	0.952	0.944	–	–
Metric	972.546	625	<0.001	0.050	0.973	0.969	0.021	−0.017
Scalar	1126.085	700	<0.001	0.052	0.967	0.967	−0.006	0.002

## Data Availability

Data are not available due to ethical restrictions.

## Appendix A

**Dimension**	**Items (Teachers)**	**Items (Parents)**	**Items (Students)**
Integration of Contextual and Cultural Knowledge	D_1. I value that my students learn about both Mapuche and Chilean culture. D_8. Learning based on the local context improves students’ understanding of what is taught in school. D_9. It is crucial to include Mapuche cultural knowledge in the school curriculum. D_10. The link between the school and the Mapuche community facilitates a better understanding and dialogue of knowledge.	P_1. I value learning about both the Mapuche culture and the Chilean culture. P_8. Learning from the local context improves understanding of what the school teaches students. P_9. It is crucial to include Mapuche cultural knowledge in the school curriculum. P_10. The link between school and community facilitates the dialogue of knowledge.	E_1. I value learning about both the Mapuche culture and the Chilean culture. E_8. I prefer that school activities include topics from the Mapuche culture. E_9. I like it when we learn about the traditions and celebrations of the Mapuche community at school. E_10. I like it when we use examples from the Mapuche community to understand the subjects. E_11. I enjoy classes that connect what I learn in school to my life at home.
School-Community Linkage and Knowledge Mobilization	D_11. It is essential to involve Mapuche families in the cultural formation of students. D_12. Rural schools promote more Mapuche cultural practices than urban ones. D_13. The school should foster a sense of belonging between Mapuche families and the educational community. D_14. The active link between family, school and community is key to preserving and strengthening the Mapuche culture.	P_11. It is essential to involve families in the cultural formation of students. P_12. Rural schools promote more Mapuche cultural practices than urban ones. P_13. The school should foster a sense of belonging between families and the educational community. P_14. The family–school–community link is key to preserving the Mapuche culture.	E_12. It is important to me that my parents come to school for cultural activities. E_13. I enjoy when activities at school involve the Mapuche community.
Equal Treatment and Recognition of Cultural Diversity	D_15. There should be no differences in the treatment between Mapuche and non-Mapuche students in the classroom. D_16. Equal treatment should recognize the cultural differences of all students. D_17. In school, all cultures must be respected equally. D_18. The Mapuche culture is equally respected in the teachings within the school. D_19. It is important that Mapuche students can express their cultural identity freely within the school. D_20. Mapuche students have the same opportunities to express their cultural identity.	P_15. There should be no differences in the treatment of Mapuche and non-Mapuche students in education. P_16. Indifferentiation in treatment must respect the diversity of students. P_17. The school promotes an environment where all cultures are equally respected. P_18. The Mapuche culture is equally respected in school education. P_19. It is important that Mapuche students are able to express their cultural identity at school. P_20. Mapuche students have the same opportunities to express their cultural identity.	E_14. I feel treated the same as my classmates at school. E_15. It’s good that all students can share about their cultures. E_16. Teachers value the different cultures of all students.
Cultural Identity and Resistance to Homogenization	D_22. The school must avoid the folklorization of Mapuche cultural ceremonies, guaranteeing their respect and depth. D_23. Respect for Mapuche values should be a central aspect in the educational approach of the school. D_24. Mapuche values should be integrated into the school curriculum to strengthen cultural identity.	P_22. The school must avoid the folklorization of Mapuche cultural ceremonies. P_23. Respect for Mapuche values should be a central element in education. P_24. Mapuche values should be integrated into the school curriculum to strengthen cultural identity.	E_17. I’m proud of my culture and I like to learn about it. E_18. I would like to learn more about Mapuche traditions at school. E_21. I want to learn more about the traditions of Mapuche families.
Intercultural Education and Deepening of Traditional Knowledge	D_25. Teachers should be more committed to teaching Mapuche cultural content in the classroom. D_26. The teaching of Mapuzugun should be a daily school practice. D_27. The school should incorporate more traditional Mapuche knowledge into the school curriculum. D_28. Education must promote a balance between traditional academic knowledge and the knowledge of the Mapuche culture. D_29. It is important that teachers have a solid knowledge of the Mapuche territory and cultural traditions. D_30. The school promotes the learning of Mapuche history and traditions as part of the comprehensive education of students.	P_25. Teachers should be more committed to teaching Mapuche cultural content. P_26. The teaching of Mapuzugun must be practical and contextualized. P_27. The school should incorporate more traditional knowledge into the school curriculum. P_28. Education should promote a balance between Western and Mapuche knowledge. P_29. The teacher should have a solid knowledge of the Mapuche territory and culture. P_30. The school promotes the learning of Mapuche history and traditions.	E_19. I think it is important to preserve the Mapuche language and its culture in school. E_20. It is important to know what the Mapuche know about plants and animals. E_22. I like it when teachers teach us about Mapuche stories.
Participation and Cultural Ceremonies	D_31. Mapuche cultural ceremonies should be an integral part of school activities. D_32. It is important for the school to hold ceremonies such as the We Txipantu. D_34. Participation in cultural ceremonies should involve the entire school community. D_35. School ceremonies reflect Mapuche cultural authenticity. D_36. School cultural activities should encourage active community participation.	P_31. Cultural ceremonies should be an integral part of school activities. P_32. It is important for the school to hold ceremonies such as the We Txipantu.P_34. Participation in cultural ceremonies should involve the entire school community. P_35. School ceremonies reflect Mapuche cultural authenticity. P_36. School cultural activities encourage community participation.	E_23. I like to participate in cultural ceremonies at school. E_24. I think it’s important that we all celebrate Mapuche cultural traditions together at school. E_25. Cultural ceremonies at school help me learn more about my cultural heritage.
